# Prevalence of lymphopenia in the American population: Insights from demographic, BMI, and lifestyle factors

**DOI:** 10.1371/journal.pone.0312540

**Published:** 2024-11-04

**Authors:** Wenchi Xie, Landie Ji, Landan Kang, Qian Li, Dan Luo, Qingquan He, Jie Mei

**Affiliations:** 1 College of Clinical Medicine, Southwest Medical University, Luzhou, Sichuan, China; 2 College of Clinical Medicine, University of Electronic Science and Technology of China, Chengdu, China; 3 Department of Obstetrics and Gynaecology, Sichuan Provincial People’s Hospital, University of Electronic Science and Technology of China, Chengdu, Sichuan, China; Children’s National Hospital, George Washington University, UNITED STATES OF AMERICA

## Abstract

**Objective:**

To determine the difference in the prevalence of lymphopenia in the American population according to demographic characteristics, body mass index (BMI) and living habits.

**Methods:**

A total of 33,365 participants aged over 1 were included in the 2009–2018 National Health and Nutrition Survey (NHANES). All analyses used weighted samples and considered the layering and clustering of the design.

**Results:**

Using white participants as a reference, the prevalence of lymphopenia in Mexican-American participants was significantly lower than that of white participants (*P* = 0.018). There was no significant difference in the prevalence of lymphopenia between black participants (*P* = 0.376) and white participants. The prevalence of lymphopenia was 1.81% (95%CI, 1.53%-2.10%) for white participants, 1.08% (95%CI, 0.78%-1.39%) for black participants, and 0.42% (95%CI, 0.17%-0.68%) for Mexican-American participants. The prevalence of lymphopenia increases with age, reaching a peak of 6.84% among elderly participants aged 75 and above. In terms of the gender difference, the prevalence of lymphopenia in men is significantly higher than that in women (*P*<0.001). Individuals who smoke (*P*<0.001), consume alcohol (*P* = 0.032), engage in regular exercise (*P* = 0.031), have sleep disorders (*P*<0.001) and those classified as having an unhealthy weight (*P*<0.001) had a higher average lymphocyte count. The prevalence of lymphopenia in participants with sleep disorders is significantly higher than those without sleep disorders (*P* = 0.014). However, no significant differences were observed among the classification variables of smoking, drinking, exercise, and BMI.

**Conclusion:**

In the diagnosis and treatment of lymphopenia, clinicians should consider the influence of factors such as race, gender, age, sleep disorders, and other unhealthy lifestyle habits to improve the accuracy of diagnosis and treatment, thereby reducing the high mortality risk associated with lymphopenia. Consequently, we propose a novel perspective that the diagnosis and treatment of lymphopenia should be tailored to the lymphocyte levels of specific subpopulations, rather than applying a generalized approach.

## Introduction

Lymphocytes play a crucial role in the immune system, aiding in the prevention, progression, and management of various diseases, including cancer, inflammation, autoimmune disorders, and infectious diseases [1]. Lymphopenia, defined as a lymphocyte count below 1.0×10^9^ cells/L [2–4], can result from primary conditions such as congenital immunodeficiency diseases or secondary factors, including malnutrition, alcoholism, medication, malignancies, systemic autoimmune diseases, and infections [5–9].

Prior investigations have revealed a strong association between lymphopenia, increased susceptibility to infections, and elevated mortality risks [2, 10]. For example, a 2018 study encompassing 98,344 participants from the general population in Denmark highlighted the heightened infection risk associated with lymphopenia [11]. Another study of 31,178 participants surveyed during the National Health and Nutrition Examination Survey from 1999 to 2010 demonstrated that individuals with lymphopenia, particularly when combined with other immune and hematological abnormalities, faced a significantly elevated mortality risk [2]. Furthermore, a prospective cohort study involving 108,135 individuals from the general population in Denmark in 2020 established that lymphopenia correlated with an increased risk of all-cause and cause-specific mortality [12]. Recent research has also investigated the correlation between COVID-19 severity and lymphopenia. Researchers identified that lymphopenia was a good predictor for the severity and recovery of COVID-19. Particularly, lymphopenia was associated with inflammatory markers, grades of pneumonia severity and prolonged hospitalization. Normalization of lymphocyte count indicated recovery of COVID-19 [13, 14].

In the past, studies have found that lymphopenia in COVID-19 patients is associated with factors such as male gender, age > 55 years, and obesity [15, 16]. Notably, no cross-sectional study to date has comprehensively explored the demographic characteristics, BMI, and lifestyle factors related to lymphopenia. Thus, to aid in better clinical diagnosis and treatment and to reduce the high mortality risk associated with lymphopenia, the aim of this study is to elucidate the prevalence patterns of lymphopenia within a large, ethnically diverse population. We seek to investigate the potential influence of factors such as age, gender, race, BMI, and lifestyle habits, including smoking, on lymphopenia’s prevalence.

## Materials and methods

### Study population

NHANES, the National Health and Nutrition Examination Survey, is a comprehensive research program designed to evaluate the health and nutritional status of individuals, both adults and children, across the United States. This survey employs a range of methods, including interviews, medical examinations, and laboratory assessments, to offer a snapshot of the nutritional and health profile of the general U.S. population. In-depth methodologies are accessible via the NHANES website (http://www.cdc.gov/nchs/nhanes.htm). NHANES was approved by the National Center for Health Statistics research ethics review board and that all participants provided written informed consent. The present study constitutes a cross-sectional analysis encompassing data from five NHANES survey cycles spanning the years 2009 to 2018. Over this period, the survey comprised a total of 47,715 participants aged one year and above. Exclusions were made for 1,971 individuals who did not undergo routine blood tests and an additional 12,379 individuals with missing data, resulting in a dataset of 33,365 participants with complete blood cell count and associated variables. The collection of routine blood measurements involved obtaining 5 ml samples of whole blood from participants through venous puncture. Blood collection occurred after a minimum fasting period of 8 hours and included anticoagulation with K3EDTA. Blood analysis was carried out using the Coulter MAXM counter, which was employed until 2013–2014 when it was replaced by the Beckman Coulter DXH 800 for blood routine measurements in the state of Ryida.

### Variable

Demographic data encompass variables such as age, race, and gender. Within this context, age is treated as a continuous variable and is subjected to a detailed, multi-tiered analysis as part of the NHANES assessment. Gender is categorized into two groups: male and female. Race is further categorized into distinct groups, including Mexican-Americans, non-Hispanic white people (hereinafter referred to as white people), non-Hispanic black people (hereinafter referred to as black people), and other Hispanics, among others. As for lifestyle information, it covers aspects such as smoking, sleep disorders, drinking, and physical activity. Within this framework, individuals designated as existing smokers are those who have smoked 100 or more cigarettes during the survey and/or are currently smoking either on a daily or occasional basis during the survey. Sleep disorders are defined based on responses to specific NHANES questions found in the sleep disorder module SLQ060 and SLQ050, which inquire if respondents have ever been informed by a doctor or healthcare professional about having a sleep disorder, or if they have ever told a healthcare professional about experiencing sleep disorder-related difficulties, with affirmative responses indicating the presence of sleep disorders [17]. Alcohol consumption is self-reported, and the definition of healthy levels is in accordance with the dietary guidelines in the United States. This entails one or fewer drinks per day for women and two or fewer drinks per day for men (with one U.S. drink containing 14 grams of ethanol). In this study, individuals classified as drinkers are considered women who consume more than one drink per day and men who consume more than two drinks per day [18, 19]. Regarding physical activity, data is analyzed in line with the guidelines of the World Health Organization. It is converted into metabolic equivalent (MET) minutes of moderate to strenuous physical activity per week. Participants are categorized based on whether their self-reported results meet the recommended criteria, which involve achieving 600 MET minutes or more per week, equivalent to 150 minutes of moderate-intensity activity or 75 minutes of high-intensity physical activity [20]. In this context, individuals meeting or exceeding this threshold are defined as exercisers. For BMI, height and weight are self-reported. BMI is calculated by dividing the subject’s weight (in kilograms) by the square of their height (in square meters). Healthy weight is defined within the BMI range of 18.5 to 24.9 [21].

### Statistical methods

All analyses in this study were performed using weighted samples. NHANES database releases data every two years from 2009 to 2018. To provide estimates for the entire 10 years, a 10-year, weight-variable sample was created by taking one fifth for the 2-year weight for each person who was sampled in 2009 to 2018. To ensure that the estimates derived are representative of the entire U.S. population, a stratification and clustering approach was employed, enabling a differential analysis of variable outcomes. Additionally, an examination of missing samples was conducted, considering race and age. Weighted linear regression was employed to adjust the averages of fundamental hematological variables for the major racial categories (black, white, Mexican-American) with respect to age and gender. Furthermore, weighted linear regression was applied to adjust the average leukocyte and lymphocyte counts of participants categorized by lifestyle habits and BMI, while taking into account age and gender. In order to assess the prevalence of lymphopenia, a weighted multivariate linear regression analysis was conducted. This analysis involved adjustments for age, gender, race, lifestyle habits, and BMI. All the aforementioned analyses were executed using the R software package (http://www.R-project.org). Chi-square tests were used to compare groups and assess significance. In the context of multiple comparisons, the threshold for statistical significance was set at *P* < 0.05.

## Results

### Missing samples and basic hematological variables

A total of 33,365 participants with valid hematology indicators in this study were considered to represent the United States’ population, which totals 252 million individuals. For the 12,379 participants with missing hematology index data, a detailed analysis was carried out, considering their race and age, which was categorized as follows: ≤5 years old, 6–17 years old, and ≥18 years old. Of the total participants, 24.8% were black, 39.6% were Mexican-Americans, and 32.3% were white. In terms of age distribution, children aged 5 and under accounted for 35.2%, children aged 6–17 accounted for 32.3%, and participants aged 18 and above represented 32.5% of the sample. To account for variations in the average basic hematological variables across major racial categories (black, white, and Mexican-Americans), weighted linear regression was employed, adjusting for age and gender. White participants were used as a reference group for differential analysis. When compared to white participants, black participants exhibited lower average leukocyte count (with an average difference of 0.64×10^9^ cells/L, *P*<0.001), lower average neutrophil count (0.65×10^9^ cells/L, *P*<0.001), and lower average hemoglobin count (0.91×10^9^ cells/L, *P*<0.001). However, they had a higher average platelet count (5.46×10^9^ cells/L, *P* = 0.004). The average lymphocyte count did not significantly differ from that of white participants (*P* = 0.179). In contrast, when compared to white participants, Mexican-American participants had a higher average leukocyte count (0.24×10^9^ cells/L, *P* = 0.004), a higher average neutrophil count (0.13×10^9^ cells/L, *P* = 0.022), and a higher average lymphocyte count (0.12×10^9^ cells/L, *P*<0.001). They exhibited a lower average hemoglobin count (0.21×10^9^ cells/L, *P*<0.001), with no significant difference in the average platelet count (*P* = 0.118) (as shown in **Table 1**). Compared to black participants, Mexican-American participants had a higher average leukocyte count (0.88×10⁹ cells/L, *P*<0.001), a higher average neutrophil count (0.79×10⁹ cells/L, *P*<0.001), and a higher hemoglobin count (0.69×10⁹ cells/L, *P*<0.001). There were no significant differences in average lymphocyte count or average platelet count (*P*>0.05).

**Table 1:** Mean hematologic values, by age, sex, and ethnic group*.

Sex and Age Group	Black Participants					
	Participants, n	Leukocyte Count, ×10^9^ cells/L	Neutrophil Count, ×10^9^ cells/L	Lymphocyte Count, ×10^9^ cells/L	Hemoglobin Level, g/L	Platelet Count, ×10^9^ cells/L
All	5613	6.6 (6.5–6.7)^ƚ^	3.5 (3.5–3.6)^ƚ^	2.3 (2.3–2.4)^ƚ^	13.2 (13.1–13.2)^ƚ^	254 (250.5–257.6)^§^
Male <18	1066	6.3 (6.2–6.5)^ƚ^	2.9 (2.7–3)^ƚ^	2.6 (2.5–2.7)^Ψ^	13 (12.9–13.1)^ƚ^	281.3 (274.5–288.1)^Ψ^
Male > = 18	1728	6.5 (6.3–6.6)^ƚ^	3.6 (3.5–3.7)^ƚ^	2.1 (2.1–2.2)^ƚ^	14.4 (14.3–14.4)^ƚ^	221.6 (218.4–224.8)^Ψ^
Female <18	982	6.8 (6.6–6.9)^ƚ^	3.2 (3.1–3.3)^ƚ^	2.8 (2.7–2.9)^Ψ^	12.4 (12.3–12.5)^ƚ^	291 (285.3–296.6)^§^
Female > = 18	1837	6.8 (6.7–6.9)^ƚ^	3.8 (3.7–3.9)^ƚ^	2.3 (2.2–2.3)^ƚ^	12.5 (12.4–12.6)^ƚ^	261 (256.3–265.7)^Ψ^
**Sex and Age Group**	**White Participants**					
	**Participants, n**	**Leukocyte Count, ×10**^**9**^ **cells/L**	**Neutrophil Count, ×10**^**9**^ **cells/L**	**Lymphocyte Count, ×10**^**9**^ **cells/L**	**Hemoglobin Level, g/L**	**Platelet Count, ×10**^**9**^ **cells/L**
All	9433	7.2 (7.1–7.3)	4.2 (4.2–4.3)	2.2 (2.1–2.2)	14.2 (14.1–14.2)	242.2 (240.2–244.2)
Male <18	1144	7.1 (6.9–7.2)	3.5 (3.4–3.6)	2.7 (2.6–2.8)	13.7 (13.6–13.8)	274.3 (269.4–279.2)
Male > = 18	3572	7.2 (7.1–7.3)	4.3 (4.2–4.4)	2 (2–2)	15.1 (15.1–15.2)	222.6 (220.1–225.1)
Female <18	1021	7.4 (7.2–7.6)	3.8 (3.6–4)	2.8 (2.7–2.9)	13.3 (13.2–13.4)	280 (275.7–284.3)
Female > = 18	3696	7.3 (7.2–7.4)	4.4 (4.3–4.5)	2.1 (2.1–2.1)	13.6 (13.5–13.6)	247.2 (245–249.4)
**Sex and Age Group**	**Mexican-American Participants**					
	**Participants, n**	**Leukocyte Count, ×10**^**9**^ **cells/L**	**Neutrophil Count, ×10**^**9**^ **cells/L**	**Lymphocyte Count, ×10**^**9**^ **cells/L**	**Hemoglobin Level, g/L**	**Platelet Count, ×10**^**9**^ **cells/L**
All	4521	7.5 (7.4–7.7)^§^	4.3 (4.2–4.4)^¶^	2.5 (2.4–2.5)^ƚ^	13.9 (13.8–14)^ƚ^	255.3 (252.1–258.5)^Ψ^
Male <18	1045	7.6 (7.5–7.7)^ƚ^	3.8 (3.7–3.9)^ƚ^	2.9 (2.8–2.9)^§^	13.6 (13.5–13.7)^Ψ^	275.1 (269.8–280.4)^Ψ^
Male > = 18	1213	7.4 (7.2–7.6)^¶^	4.4 (4.2–4.5)^Ψ^	2.2 (2.1–2.3)^ƚ^	15.3 (15.2–15.4)^§^	229.5 (225.2–233.8)^§^
Female <18	1009	7.7 (7.5–7.9)^¶^	4 (3.8–4.1)^Ψ^	2.9 (2.8–3)^Ψ^	13 (12.9–13.1)^ƚ^	283 (278–288)^Ψ^
Female > = 18	1254	7.6 (7.4–7.8)^§^	4.6 (4.4–4.7)^Ψ^	2.3 (2.2–2.3)^ƚ^	13.1 (13–13.1)^ƚ^	258.8 (254.9–262.7)^Ψ^

* Means (95% CIs) from a weighted analysis adjusted for age and sex are given unless otherwise indicated.

^ƚ^ *P*<0.001 compared with white participants.

^Ψ^ *P*<0.05 compared with white participants.

^**§**^ *P*<0.001 to 0.01 compared with white participants.

^**¶**^ *P*<0.01 to 0.05 compared with white participant.

**Fig 1:**
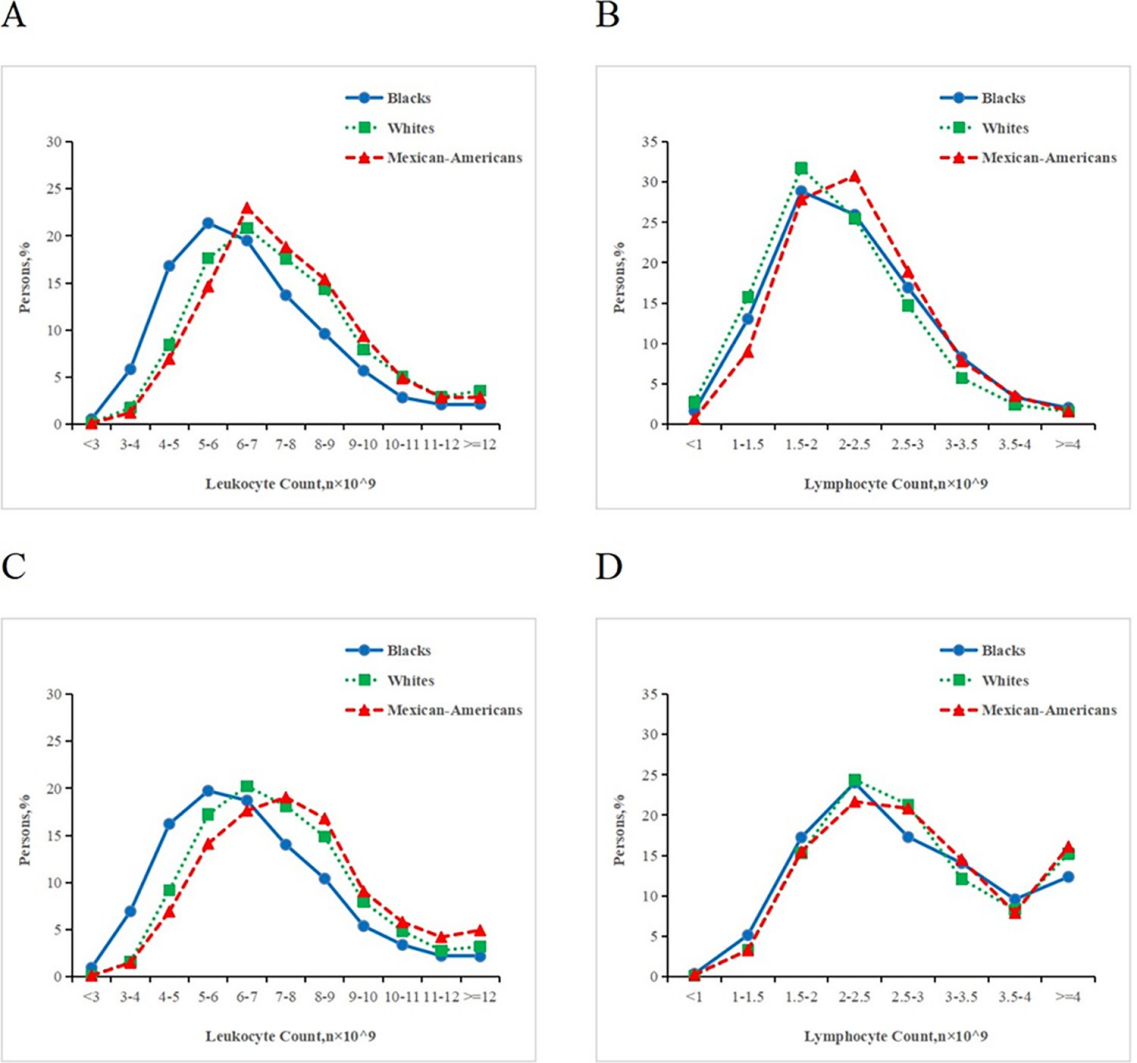
Distributions of leukocytes and lymphocytes in participants of three races. (A-B) Distributions of leukocyte and lymphocyte counts in persons aged 18 years or older from 3 ethnic groups. (C-D) Distributions of leukocyte and Lymphocyte counts in persons less than 18 years old from 3 ethnic groups.

**Fig 1** illustrates the distribution of leukocytes and lymphocytes in two age groups: participants under 18 and participants aged 18 and above. In the group of participants aged 18 and above, black participants had a decrease of approximately 1.0×10^9^ cells/L in leukocyte count when compared to white participants, with little difference in lymphocyte count. Mexican-American participants exhibited a similar leukocyte count compared to white participants, with a slight increase in lymphocyte count. In the group of participants under 18, the distribution of leukocyte and lymphocyte counts for black participants closely resembled that of white participants. Mexican-Americans, when compared to white participants, had a slight increase in leukocyte count and only a minor difference in lymphocyte count.

### Effects of living habits and BMI on average leukocyte and lymphocyte count

The effects of various living habits and BMI on average leukocyte and lymphocyte counts were examined. We adjusted these counts according to age and gender, and the results are presented in **Table 2**. Overall, for the average leukocyte count, smokers exhibited higher counts (an average difference of 0.76×10^9^ cells/L, *P*<0.001), as did drinkers (0.44×10^9^ cells/L, *P*<0.001), participants with sleep disorders (0.36×10^9^ cells/L, *P*<0.001), exercisers (0.49×10^9^ cells/L, *P*<0.001), and unhealthy weight participants (0.62×10^9^ cells/L, *P*<0.001). Concerning the average lymphocyte count, smokers had higher counts (0.14×10^9^ cells/L, *P*<0.001), as did drinkers (0.06×10^9^ cells/L, *P* = 0.032), participants with sleep disorders (0.07×10^9^ cells/L, *P*<0.001), exercisers (0.08×10^9^ cells/L, *P* = 0.031), and unhealthy weight participants (0.24×10^9^ cells/L, *P*<0.001). From a racial perspective, the impact of these factors on the average leukocyte count was as follows: smoking had the greatest impact on white participants (0.81×10^9^ cells/L, *P*<0.001), followed by black participants (0.68×10^9^ cells/L, *P*<0.001), with Mexican-American participants experiencing a relatively smaller impact (0.55×10^9^ cells/L, *P*<0.001). Drinking had the greatest impact on black participants (0.49×10^9^ cells/L, *P* = 0.007), a greater impact on white participants (0.46×10^9^ cells/L, *P*<0.001), and no significant impact on Mexican-American participants (*P* = 0.133). Sleep disorders had the greatest impact on white participants (0.42×10^9^ cells/L, *P*<0.001), while having no significant impacts on black (*P* = 0.053) and Mexican-American participants (*P* = 0.192). Exercise had an impact on the increase in the average leukocyte count of white participants (0.53×10^9^ cells/L, *P*<0.001), with no significant impact on black (*P* = 0.296) and Mexican-American participants (*P* = 0.420). Unhealthy weight had the greatest impact on Mexican-American participants (0.70×10^9^ cells/L, *P*<0.001), followed by white participants (0.63×10^9^ cells/L, *P*<0.001), and had a smaller impact on black participants (0.54×10^9^ cells/L, *P*<0.001). Regarding the average lymphocyte count, smoking had the greatest impact on black participants (0.21×10^9^ cells/L, *P*<0.001), a lesser impact on white participants (0.16×10^9^ cells/L, *P*<0.001), and the smallest influence on Mexican-American participants (0.08×10^9^ cells/L, *P* = 0.008). Drinking had an impact on the increase in the average lymphocyte count of white participants (0.08×10^9^ cells/L, *P* = 0.017), but had no significant impact on black participants (*P* = 0.208) and Mexican-American participants (*P* = 0.911). Sleep disorders had the greatest impact on the increase in the average lymphocyte count of white participants (0.09×109 cells/L, P<0.001), a greater impact on black participants (0.07×10^9^ cells/L, *P* = 0.037), and no significant impact on Mexican-American participants (*P* = 0.385). Exercise had no significant impact on the average lymphocyte count for participants of all three races (white: *P* = 0.064, black: *P* = 0.161, Mexican-American: *P* = 0.820). Unhealthy weight had the greatest impact on the increase in the average lymphocyte count for Mexican-American participants (0.27×10^9^ cells/L, *P*<0.001), followed by black participants (0.24×10^9^ cells/L, *P*<0.001), and had a relatively smaller impact on white participants (0.22×10^9^ cells/L, *P*<0.001).

**Table 2:** Comparative mean leukocyte and lymphocyte counts in smokers and nonsmokers aged 18 years or older.

Variable Leukocyte count, ×10^9^ cells/L	Smokers	Nonsmokers	Mean Difference *	*P* Value^˥^
Black people	7.0 (6.8–7.1)	6.4 (6.3–6.5)	0.68 (0.59–0.77)	<0.001
White people	7.6 (7.5–7.7)	6.9 (6.8–7.0)	0.81 (0.75–0.87)	<0.001
Mexican-Americans	7.7 (7.6–7.9)	7.3 (7.2–7.5)	0.55 (0.47–0.89)	<0.001
**Lymphocyte count, ×10**^**9**^ **cells/L**				
Black people	2.3 (2.2–2.3)	2.2 (2.1–2.2)	0.21 (0.18–0.24)	<0.001
White people	2.1 (2.1–2.2)	2.0 (2.0–2.1)	0.16 (0.14–0.18)	<0.001
Mexican-Americans	2.3 (2.2–2.3)	2.2 (2.2–2.3)	0.08 (0.05–0.11)	= 0.008
Comparative Mean Leukocyte and Lymphocyte Counts in Drinkers and Nondrinkers Aged 18 Years or Older.				
**Variable Leukocyte count, ×10**^**9**^ **cells/L**	**Drinkers**	**Nondrinkers**	**Mean Difference** *	***P* Value** ^**˥**^
Black people	6.8 (6.7–6.9)	6.6 (6.4–6.8)	0.49 (0.33–0.65)	= 0.007
White people	7.4 (7.2–7.5)	7.0 (6.8–7.1)	0.46 (0.38–0.54)	<0.001
Mexican-Americans	7.5 (7.4–7.7)	7.2 (6.9–7.4)	0.21 (0.08–0.34)	= 0.133
**Lymphocyte count, ×10** ^**9**^ **cells/L**				
Black people	2.3 (2.2–2.3)	2.2 (2.1–2.2)	0.07 (0.02–0.12)	= 0.208
White people	2.1 (2.1–2.1)	2.0 (1.9–2.0)	0.08 (0.05–0.11)	= 0.017
Mexican-Americans	2.3 (2.2–2.3)	2.1 (2.1–2.2)	0.01 (0.00–0.05)	= 0.911
Comparative Mean Leukocyte and Lymphocyte Counts in Participants with Sleep Disorders and Participants without Sleep Disorders Aged 16 Years or Older.				
**Variable Leukocyte count, ×10**^**9**^ **cells/L**	**Participants with sleep disorders**	**Participants without sleep disorders**	**Mean Difference** *	***P* Value** ^**˥**^
Black people	2.3 (2.2–2.3)	2.2 (2.2–2.2)	0.19 (0.10–0.28)	= 0.053
White people	2.1 (2.0–2.1)	2.0 (2.0–2.1)	0.42 (0.37–0.47)	<0.001
Mexican-Americans	2.2 (2.1–2.3)	2.3 (2.2–2.3)	0.18 (0.05–0.31)	= 0.192
**Lymphocyte count, ×10**^**9**^ **cells/L**				
Black people	2.3 (2.2–2.3)	2.2 (2.2–2.2)	0.07 (0.04–0.10)	= 0.037
White people	2.1 (2.0–2.1)	2.0 (2.0–2.1)	0.10 (0.08–0.12)	<0.001
Mexican-Americans	2.2 (2.1–2.3)	2.3 (2.2–2.3)	0.04 (0.00–0.08)	= 0.385
Comparative Mean Leukocyte and Lymphocyte Counts in Exercisers and Non-exercisers Aged 12 Years or Older.				
**Variable Leukocyte count, ×10**^**9**^ **cells/L**	**Exercisers**	**Non-exercisers**	**Mean Difference** *	***P* Value** ^**˥**^
Black	6.6 (6.4–6.9)	6.5 (6.2–6.8)	0.19 (0.02–0.36)	= 0.296
White	7.3 (7.1–7.4)	6.8 (6.6–7.1)	0.53 (0.40–0.66)	<0.001
Mexican American	7.5 (7.3–7.7)	7.5 (7.1–7.9)	0.15 (0.00–0.33)	= 0.420
**Lymphocyte count, ×10**^**9**^ **cells/L**				
Black people	2.3 (2.2–2.3)	2.2 (2.1–2.3)	0.08 (0.03–0.13)	= 0.161
White people	2.1 (2.0–2.2)	2.1 (2.0–2.1)	0.10 (0.05–0.15)	= 0.064
Mexican-Americans	2.2 (2.2–2.3)	2.3 (2.2–2.4)	0.01 (0.00–0.07)	= 0.820
Comparative Mean Leukocyte and Lymphocyte Counts in Unhealthy Weight Participants and Healthy Weight Participants Aged 2 Years or Older.				
**Variable Leukocyte count, ×10**^**9**^ **cells/L**	**Unhealthy Weight Participants**	**Healthy Weight Participants**	**Mean Difference** *	***P* Value** ^**˥**^
Black people	6.8 (6.7–6.9)	6.2 (6.0–6.4)	0.54 (0.47–0.61)	<0.001
White people	7.4 (7.3–7.5)	6.8 (6.7–6.9)	0.63 (0.57–0.69)	<0.001
Mexican-Americans	7.7 (7.5–7.8)	7.0 (6.9–7.1)	0.70 (0.63–0.77)	<0.001
**Lymphocyte count, ×10**^**9**^ **cells/L**				
Black people	2.4 (2.3–2.4)	2.2 (2.1–2.2)	0.24 (0.00–0.49)	<0.001
White people	2.2 (2.1–2.2)	2.0 (2.0–2.1)	0.22 (0.20–0.24)	<0.001
Mexican-Americans	2.4 (2.4–2.5)	2.3 (2.2–2.3)	0.27 (0.25–0.29)	<0.001

* The respective numbers of participants who smoked and those who did not smoke were 1468 and 2763 for black, 3662 and 4320 for white, and 848 and 2262 for Mexican-American participants. The respective numbers of participants who were drinkers and those who did not drink were 459 and 3042 for black, 1092 and 6085 for white, and 326 and 2061 for Mexican-American participants. The respective numbers of participants who had sleep disorders and who did not had sleep disorders were 926 and 2877 for black, 2394 and 5110 for white, and 469 and 2214 for Mexican- American participants. The respective numbers of participants who were exercisers and who were not exercisers were 189 and 376 for black, 384 and 1070 for white, and 154 and 307 for Mexican-American participants. The respective numbers of participants who had unhealthy weight and who did not had healthy weight were 3942 and 1504 for black, 6348 and 2835 for white, and 3192 and 1134 for Mexican- American participants.

^˥^ Comparisons of the means between smokers and nonsmokers, drinkers and nondrinkers,participants with sleep disorders and participants without sleep disorders, exercisers and non-exercisers, and unhealthy weight participants and healthy weight participants, according to ethnicity and adjusted for age and sex.

### Prevalence of lymphopenia

**Fig 2:**
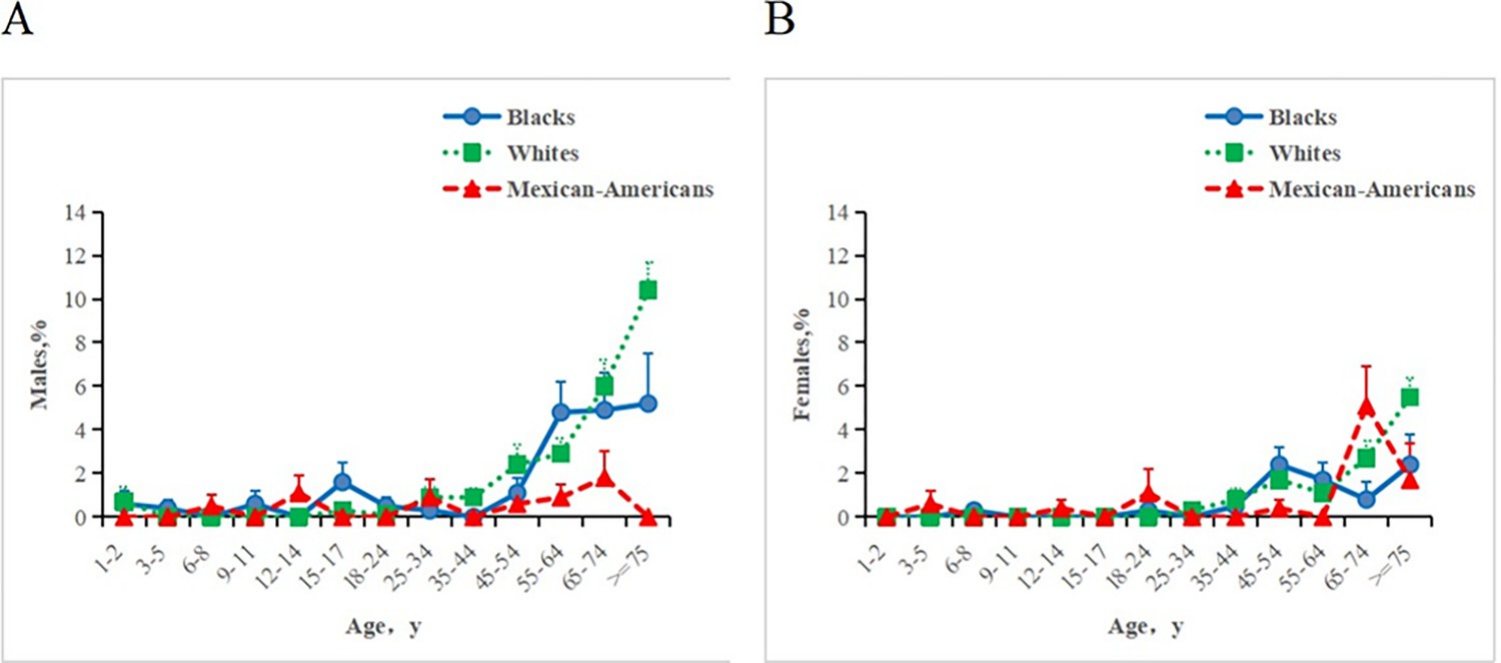
Percentage of overall male and female participants with lymphocyte counts of less than 1.0×10^9^ cells/L. The error bars refer to the standard errors.

Weighted multivariate linear regression analysis was employed to estimate the prevalence of age, sex, and race-adjusted lymphopenia (≤1.0×10^9^ cells/L) as presented in **Table 3**. Among all participants, 334 individuals had lymphocyte counts below 1.0×10^9^ cells/L. The prevalence rate was 1.4% (95%CI, 1.2%-1.6%), suggesting that the total number of cases of lymphopenia in the United States was approximately 2.41 million. From a racial perspective, Mexican-American participants were less likely to experience lymphopenia (*P* = 0.018) compared to white participants. However, black participants did not exhibit a significant difference (*P* = 0.376). The prevalence of lymphopenia was highest among white participants, at 1.81% (95%CI, 1.53%-2.10%), followed by black participants at 1.08% (95%CI, 0.78%-1.39%), with Mexican-American participants having the lowest prevalence rate of 0.42% (95%CI, 0.17%-0.68%). Regarding gender, male participants were more susceptible to lymphopenia (*P*<0.001), with prevalence rates of 1.81% (95%CI, 1.47%-2.16%) and 1.08% (95%CI, 0.86%-1.30%) for men and women, respectively. Among white and black participants, the prevalence rate of lymphopenia was higher in men than in women. However, among Mexican-American participants, the prevalence rate among women was slightly higher than that among men. For black male participants, the prevalence rate was 2.31%, compared to 1.33% for women. Among white participants, the rate was 1.40% for men and 0.82% for women, while among Mexican-American participants, it was 0.40% for men and 0.45% for women. In terms of age, the prevalence of lymphopenia was less than 1.00% before the age of 44, showing an overall upward trend. Among elderly participants aged 75 and above, the prevalence rate increased significantly, reaching a maximum of 6.84%. In general (as shown in **Fig 2**), among male participants, the prevalence of lymphopenia was lower for Mexican-American participants compared to white and black participants. The difference in prevalence was not substantial before the age of 44. When compared with white participants, the prevalence of lymphopenia in black participants was significantly lower after the age of 64, but there was no significant difference before the age of 64. The same trend was observed among female participants. Notably, the prevalence rate of lymphopenia in Mexican-American participants aged 65–74 significantly exceeded that of black and white participants, possibly due to certain errors. Furthermore, we analyzed the interaction between the three variables of race, age, and gender and found that there was no significant interaction between race and sex, age and race, and race and age (*P*>0.05). For smokers and nonsmokers, drinkers and nondrinkers, participants with sleep disorders and without sleep disorders, exercisers and non-exercisers, as well as healthy weight participants and unhealthy weight participants, separate weighted linear regression analyses were conducted and adjusted for race, gender, and age. The prevalence of lymphopenia was higher among participants with sleep disorders (*P* = 0.014). There were no significant differences in the prevalence between smokers and nonsmokers (*P* = 0.376), drinkers and nondrinkers (*P* = 0.504), exercisers (*P* = 0.087), and non-exercisers, as well as healthy weight participants and unhealthy weight participants (*P* = 0.085).

**Table 3:** Prevalence of a lymphocyte count less than 1.0 ×10^9^ cells/L.

Variable	Prevalence, %* (95% CI)
**Ethnicity**	
white	1.81 (1.53–2.10)
black	1.08 (0.78–1.39)
Mexican-American	0.42 (0.17–0.68)
**Sex**	
Male	1.81 (1.47–2.16)
Female	1.08 (0.86–1.30)
**Age**	
1–2	0.22 (-0.14–0.58)
3–5	0.13 (-0.02–0.29)
6–8	0.11 (-0.02–0.25)
9–11	0.11 (-0.01–0.23)
12–14	0.16 (-0.01–0.33)
15–17	0.3 (0.06–0.53)
18–24	0.16 (0–0.31)
25–34	0.53 (0.12–0.93)
35–44	0.59 (0.23–0.96)
45–54	1.7 (0.92–2.48)
55–64	2.01 (1.33–2.69)
65–74	3.98 (2.85–5.11)
> = 75	6.84 (5.49–8.2)

* Prevalence is estimated as the predicted marginals in the logistic regression analysis. The estimates for white, black, and Mexican-American participants are adjusted for age and sex.

### Distribution of lymphocyte count in patients with lymphopenia

The lymphocyte counts of 324 participants with lymphopenia were distributed in the range of (0.5–1.0)×10^9^ cells/L. Within this range, 96.2% of black participants with lymphopenia, 98.0% of white participants with lymphopenia, and 92.8% of Mexican-American participants with lymphopenia had lymphocyte counts. In the (0.5–1.0)×10^9^ cells/L range, black participants with lymphopenia represented 1.04% of the total black participants, white participants with lymphopenia accounted for 1.78% of the total white participants, and Mexican-American participants with lymphopenia represented 0.40% of the total Mexican-American participants (**Table 4**). In the category with lymphocyte counts less than 0.5×10^9^ cells/L, black participants with lymphopenia accounted for 0.04% of the total black participants, white participants with lymphopenia accounted for 0.04% of the total white participants, and Mexican-American participants with lymphopenia represented 0.03% of the total Mexican-American participants. Overall, the incidence of lymphocyte counts less than 0.5×10^9^ cells/L in participants of all three races was relatively low.

**Table 4:** Overall prevalence of participants with lymphocyte counts of 0.5–1.0 ×10^9^ cells/L.

Age Group	Black people	White people	Mexican-Americans
1–2	1 (0.3) [-0.3–0.9]	1 (0.4) [-0.4–1.1]	0 (0) [0–0]
3–5	1 (0.2) [-0.2–0.6]	0 (0) [0–0]	1 (0.3) [0–0.8]
6–8	1 (0.1) [-0.1–0.4]	0 (0) [0–0]	0 (0) [0–0]
9–11	1 (0.3) [-0.2–0.8]	0 (0) [0–0]	0 (0) [0–0]
12–14	0 (0) [0–0]	0 (0) [0–0]	3 (0.7) [0–1.6]
15–17	3 (0.9) [-0.1–1.9]	1 (0.2) [-0.1–0.5]	0 (0) [0–0]
18–24	3 (0.4) [-0.1–0.9]	1 (0.1) [-0.1–0.2]	1 (0.5) [0–1.4]
25–34	1 (0.1) [-0.1–0.4]	5 (0.6) [0–1.2]	1 (0.4) [0–1.3]
35–44	2 (0.3) [-0.1–0.7]	10 (0.8) [0.2–1.5]	0 (0) [0–0]
45–54	10 (1.8) [0.8–2.8]	23 (2) [0.8–3.1]	3 (0.5) [0–1.1]
55–64	21 (3.1) [1.5–4.8]	24 (2) [1.1–2.8]	1 (0.2) [0–0.7]
65–74	12 (2.6) [0.9–4.4]	38 (4.1) [2.7–5.4]	8 (3.6) [1.0–6.1]
> = 75	7 (2.3) [0.4–4.2]	92 (7.4) [5.9–9]	1 (0.9) [0–2.6]

## Discussion

### Causes of the lymphopenia

Congenital lymphopenia is primarily associated with certain congenital immunodeficiency diseases, such as congenital athymia, Bruton’s agammaglobulinemia, severe combined immunodeficiency disease, and ataxia capillary dilatation immunodeficiency. These conditions are largely influenced by genetic factors and heredity [22]. Another major cause of secondary lymphopenia is viral infections. For example, individuals with HIV who are co-infected with monkeypox virus and other pathogens often tend to have low CD4+ T cell counts [23]. As the duration of infection increases, natural killer cells and T cells can become exhausted, resulting in a decline in lymphocyte numbers [24]. Moreover, certain unhealthy behaviors, such as malnutrition caused by dieting or picky eating, can negatively impact the body’s immune function and may lead to physiological lymphopenia. Studies have shown that balanced nutrition and appropriate body weight are essential for maintaining normal metabolic activities and immune function. Malnourished individuals often experience a reduction in the total number of T lymphocytes and a decline in their function. Additionally, malnutrition can damage the body’s mechanical barriers, lower mucosal resistance, and lead to immune dysfunction, making individuals more susceptible to various infections [25].

### Analysis of differences in basic hematological variables

Basic hematological indicators play a guiding role in clinical diagnosis and treatment. In addition to their roles in hemostasis and thrombosis, platelets are increasingly recognized as key modulators of inflammatory responses under both sterile and infectious conditions. The interaction between platelets and leukocytes regulates the resolution of inflammation, tissue repair, and wound healing. Leukocytes, as markers of inflammation, can effectively predict patients with inflammatory diseases, particularly various types of cancer. The primary clinical impact of neutropenia is an increased susceptibility to infections, with the risk of infection closely related to the severity and duration of neutropenia. Hemoglobin also serves as a predictor of cancer, with a decline in hemoglobin levels associated with undiagnosed cancer [26–29]. In our analysis of nationally representative data, we discovered variations in basic hematological variables. Compared to white participants, black participants exhibited lower average leukocyte, neutrophil, and hemoglobin counts, but higher average platelet counts. There was no significant difference in the average lymphocyte count between black and white participants. In contrast, Mexican-American participants displayed higher average leukocyte, neutrophil, and lymphocyte counts compared to white participants, but lower average hemoglobin counts. There were no significant differences in average platelet counts.

It’s worth noting that prior research has yielded inconsistent findings regarding the lymphocyte count differences among different racial groups. Some studies found higher lymphocyte counts in black participants, which may be indicative of infection, autoimmune diseases, or certain blood disorders. However, the specific mechanisms remain unclear, but they may be related to the environment in which Black participants live. For example, they may be at a higher risk of infection due to factors such as exposure to virus-carrying mosquitoes or the consumption of contaminated water. This could lead to an active antiviral response early in the infection, resulting in an increase in lymphocyte counts [30–32]. Conversely, other studies reported no significant differences among ethnic groups [33]. In our study, we observed elevated lymphocyte counts in Mexican-American participants when compared to white participants, but this difference did not reach statistical significance among the black population.

### Analysis of the difference in prevalence of lymphopenia

In 2022, a study conducted by Noah et al. utilized multiple linear regression analysis to reveal that in adult patients hospitalized for COVID-19, lymphocyte counts decreased with age, and men exhibited lower counts than women [34]. For the general population, our study also observed the same conclusion that the prevalence of lymphopenia was increasing in age. Our research on the general population corroborated these findings, demonstrating an age-related increase in the prevalence of lymphopenia. As age advanced, the prevalence of lymphopenia rose, and this condition was more prevalent in men than women. This gender discrepancy may be attributed to hormonal influences within the body. Women typically have higher counts of CD4+T cells and B cells, along with an elevated CD4+/CD8+T cell ratio compared to men. These gender differences remain consistent from birth through old age [35]. The observed differences related to age may stem from the immaturity of the immune system in children, making them less susceptible to lymphopenia. In contrast, elderly participants were more prone to lymphopenia due to immune aging, and the prevalence increased with advancing age. This is because, with aging, the immune system’s functional decline is characterized by a shift from a naïve T-cell phenotype to a memory T-cell phenotype, a transition from a Type 1 to a Type 2 cytokine profile, humoral immune deficiencies, increased T-cell maturation rates, chronic low-grade inflammation, and many other changes [36, 37].

Previous studies have shown that patients with relapsing-remitting multiple sclerosis who had low lymphocyte counts, medium BMI(25–30), were of white ethnicity, elderly, or nonsmokers, exhibited a significantly higher incidence of lymphopenia after receiving dimethyl fumarate treatment [38]. In the general population, our study found that the prevalence of lymphopenia was notably lower in Mexican-American participants compared to white participants. Interestingly, there was no significant difference in the prevalence of lymphopenia between black participants and white participants. Past research has indicated an increased risk of abnormal CD8+ cell counts in individuals of white ethnicity, which may contribute to a significantly higher prevalence of lymphopenia [39].

Our study revealed that participants with sleep disorders had a notably high prevalence of lymphopenia, which aligns with expectations. Sleep dysfunction can compromise the body’s immune function, primarily affecting CD4+T cells and myeloid cells, and enhancing the interaction between Th17 and myeloid cells, potentially resulting in reduced lymphocyte counts. This, in turn, may elevate the risk of morbidity [40].

Prior studies have shown that smoking can increase the activation and proliferation of T cells in lymphocytes, with some changes persisting in CD8+ and CD8+ memory T cell subgroups even after quitting smoking [41]. Weight gain can impact vitamin D levels, with the resulting increase in vitamin D levels potentially inhibiting T cell proliferation and increasing cell apoptosis [42, 43]. Alcohol consumption has been associated with damage to CD4+T cell immune metabolism, hindrance of mitochondrial repair processes, and the promotion of the differentiation of CD4+T cells into an inflammatory phenotype [44, 45]. Exercise can also influence lymphocytes in the body, potentially reducing the frequency of aged klRG1+T cells and helping maintain the proportion of CD4+ naïve T cells, which is indicative of successful rather than maladaptive T cell aging [46, 47]. Exercise can also influence lymphocytes in the body, potentially reducing the frequency of aged klRG1 + T cells and helping maintain the proportion of CD4+ naïve T cells, which is indicative of successful rather than maladaptive T cell aging [48, 49]. In our study, we found that smokers, drinkers, participants with sleep disorders, exercisers, and those with unhealthy body weight had higher average lymphocyte counts. However, we did not observe a significant impact of smoking, drinking, exercise, or body weight on the prevalence of lymphopenia.

### Innovation and limitation

Our study offers a comprehensive analysis of the distribution of fundamental hematological variables across different races, genders, and age groups. Furthermore, we examine the prevalence of lymphopenia in various subpopulations categorized by race, gender, age, lifestyle factors, and BMI. Our findings reveal marked disparities in the prevalence of lymphopenia based on gender, age, and race. Additionally, we observe that sleep disorders exert a discernible influence on this prevalence. Therefore, it is advisable to approach the diagnosis and treatment of lymphopenia with consideration of specific subpopulations rather than adopting a one-size-fits-all approach. Nonetheless, it is important to acknowledge certain limitations in our study. Notably, our analysis did not incorporate factors such as dietary habits, medication usage, and the presence of chronic diseases, all of which could potentially impact lymphocyte counts and research outcomes. For example, the antiviral drug valganciclovir has been significantly associated with lymphopenia when used in excess [50]. Furthermore, the data utilized in our study is derived from the American population. Geographical attributes like climate and economic conditions may introduce some corresponding effects on the results. For example, in warmer climates where infectious diseases like malaria are more prevalent, this can have an impact on lymphocyte counts [51]. Consequently, future research endeavors may benefit from incorporating relevant hematological data from diverse geographical populations to enhance the precision and generalizability of our findings.

## Conclusion

Our analysis revealed significant disparities in the prevalence of lymphopenia within the population based on factors such as age, gender, race, living habits, and BMI. Consequently, our findings emphasize a novel perspective on the diagnosis and treatment of lymphopenia. We propose that addressing lymphopenia should involve consideration of subpopulations rather than adopting a uniform approach based on the overall population’s lymphocyte levels.
