# Correction: Prevalence of lymphopenia in the American population: Insights from demographic, BMI, and lifestyle factors

**DOI:** 10.1371/journal.pone.0316590

**Published:** 2024-12-26

**Authors:** Wenchi Xie, Landie Ji, Landan Kang, Qian Li, Dan Luo, Qingquan He, Jie Mei

There are errors in the author affiliations. The correct affiliations are as follows:

Wenchi Xie^1^, Landie Ji^2^, Landan Kang^2^, Qian Li^1^, Dan Luo^1^, Qingquan He^2^, Jie Mei^1,3^

**1** Department of Obstetrics and Gynecology, The Affiliated Hospital of Southwest Medical University, Luzhou, Sichuan, China, **2** College of Clinical Medicine, University of Electronic Science and Technology of China, Chengdu, China, **3** Department of Obstetrics and Gynaecology, Sichuan Provincial People’s Hospital, University of Electronic Science and Technology of China, Chengdu, Sichuan, China.
